# Real-time feedback control of split-belt ratio to induce targeted step length asymmetry

**DOI:** 10.1186/s12984-022-01044-0

**Published:** 2022-06-30

**Authors:** Sean Carr, Fatemeh Rasouli, Seok Hun Kim, Kyle B. Reed

**Affiliations:** 1 Department of Medical Engineering, University of South Florida, Tampa, 33620 USA; 2 Department of Mechanical Engineering, University of South Florida, Tampa, 33620 USA; 3 School of Physical Therapy and Rehabilitation Sciences, University of South Florida, Tampa, 33620 USA

**Keywords:** Split-belt, Feedback, Gait, Rehabilitation, Asymmetry, Biomechanics

## Abstract

**Introduction:**

Split-belt treadmill training has been used to assist with gait rehabilitation following stroke. This method modifies a patient’s step length asymmetry by adjusting left and right tread speeds individually during training. However, current split-belt training approaches pay little attention to the individuality of patients by applying set tread speed ratios (e.g., 2:1 or 3:1). This generalization results in unpredictable step length adjustments between the legs. To customize the training, this study explores the capabilities of a live feedback system that modulates split-belt tread speeds based on real-time step length asymmetry.

**Materials and methods:**

Fourteen healthy individuals participated in two 1.5-h gait training sessions scheduled 1 week apart. They were asked to walk on the Computer Assisted Rehabilitation Environment (CAREN) split-belt treadmill system with a boot on one foot to impose asymmetrical gait patterns. Each training session consisted of a 3-min baseline, 10-min baseline with boot, 10-min feedback with boot (6% asymmetry exaggeration in the first session and personalized in the second), 5-min post feedback with boot, and 3-min post feedback without boot. A proportional-integral (PI) controller was used to maintain a specified step-length asymmetry by changing the tread speed ratios during the 10-min feedback period. After the first session, a linear model between baseline asymmetry exaggeration and post-intervention asymmetry improvement was utilized to develop a relationship between target exaggeration and target post-intervention asymmetry. In the second session, this model predicted a necessary target asymmetry exaggeration to replace the original 6%. This prediction was intended to result in a highly symmetric post-intervention step length.

**Results and discussion:**

Eleven out of 14 participants (78.6%) developed a successful relationship between asymmetry exaggeration and decreased asymmetry in the post-intervention period of the first session. Seven out of the 11 participants (63.6%) in this successful correlation group had second session post-intervention asymmetries of < 3.5%.

**Conclusions:**

The use of a PI controller to modulate split-belt tread speeds demonstrated itself to be a viable method for individualizing split-belt treadmill training.

## Introduction

Conventional split-belt treadmill training techniques often do not consider the individuality of participants due to the use of predefined tread speed ratios, where the speed of the left belt and the speed of the right belt are constant and not equal [7, 14, 16, 22]. These ratios are applied for a period of time (split-belt training) and then the belts are returned to the same speed (tied-belt). The gait behavior retains short-term after-effects when the treadmill is returned to tied-belt. Since split belt ratios affect different participants differently, set ratios do not allow for targeted after-effects. As participants’ responsiveness to training has considerable variation, the need for individualized training strategies has become pertinent.

This study investigates the use of a real-time feedback controller focused on maintaining a desired step length asymmetry. The primary purpose of this study is to evaluate the ability of the controller to achieve target asymmetries in gait by focusing on step length and adjusting split-belt speed ratios using real-time feedback control. Additionally, we developed a model for each participant to predict second session outcomes based on first session performance. Therefore, the second purpose of the study is to determine the validity of a model that predicts intervention outcomes and individualizes training to achieve post-intervention step length asymmetry values < 3.5%.

### Split-belt treadmill training

Split-belt treadmill training is an approach taken to modify gait patterns, particularly step length asymmetries [1, 2, 5–7, 14, 16–18, 22]. The belts are set such that one is “fast” and the other is “slow.” The typical initial response to this perturbation is that the step length on the slow belt will be longer while the step length on the fast belt will be shorter [17, 20]. Upon a sudden return to a tied-belt state, the participant expresses more symmetric step lengths, opposite in direction of those induced by the split-belt intervention (i.e., after-effect) [17, 20, 22]. The after-effects imposed by various split-belt ratios between 1:1 and 1:3 have been investigated [2, 26]. These experiments demonstrated a correlation between the belt speed ratio used during the adaptation period and the severity of the step length asymmetry observed in the early adaptation period (split-belt training) and the post-adaptation period (tied-belt after-effect). In the case of post-stroke participants, the transfer of after-effects from split-belt treadmill training to ground have also been studied. The after-effect imposed by a 2:1 split-belt ratio in post-stroke participants was partially transferred from the treadmill to overground after a short-term training [19]. The limited transfer of the after-effect to overground was still observed even after a long-term (4 weeks) split-belt treadmill training [17].

The split-belt treadmill approach has also been modified to determine whether sudden or gradual deviation from tied-belt to split-belt has any effect on participant performance. Hinkel-Lipsker and Hahn [5] conducted an experiment where one group was brought from tied-belt (0.7 m/s) to 2:1 split-belt (1.4 m/s to 0.7 m/s) by an acceleration profile of 0.02 m/s^2^ every 20 strides and a second group by an acceleration profile of 10 m/s^2^. This experiment revealed a novel kinetic pattern at the hip joint of those trained with the gradual split belts. Specifically, the 10 m/s^2^ acceleration scenario resulted in decreased work at the slow hip joint. Conversely, the slower acceleration scenario resulted in little to no difference in work done between the fast and slow hip joint. This study indicates that adaptation occurs regardless of whether the treadmill belt ratio changes gradually or suddenly. The overall effects of these training approaches need to be further explored to determine their efficacy when compared to standard sudden split-belt training.

Split-belt treadmill studies have consistently aimed at revealing the relationship between gait parameters and the tread ratios used for training. They often incorporate the analysis of step length and several other spatiotemporal parameters as well as dynamic parameters, such as joint work [23]. This feedback study does not aim at understanding the effect of a new and targeted intervention on all spatiotemporal and dynamic gait parameters. Rather, it aims at determining whether a single spatial parameter (step length) can be controlled in a highly targeted manner. Confirmation of this approach will allow future investigators to determine the clinical capacity of this new intervention through analysis of its effects on more gait parameters.

### Inducing asymmetry

To evaluate our proposed method, we first induce initial step length asymmetries in healthy individuals to approximate asymmetries inherent in persons post-stroke. Commonly used strategies for inducing gait impairments are the application of weight, height, or both to participants [9, 11]. Applying a weight of 5% participant body mass is known to induce step length asymmetries as high as 7% [9]. Further, inducing a leg length discrepancy of only 3 cm has produced step length asymmetries of approximately 6% [11]. Previous research has also applied various combinations of weight and height to participants’ legs to induce asymmetries [8]. Muratagic et al. [8] found that there was no significant interaction between the effects produced by the addition of weight and height. Essentially, height and weight perturb gait independently, and their modifications add up when applied at the same time. Analysis of these results allows for the determination of which intervention is more efficient. The addition of a 5.2 cm leg length discrepancy induced approximately a 6% asymmetry, while a 4.6 kg weight only induced approximately a 2% asymmetry [8]. Thus, to create a large step length asymmetry without adding so much weight that the participant becomes exhausted, the addition of leg length is beneficial.

Exaggerating gait asymmetries facilitates the central nervous system to adapt such that the after-effects improve symmetry when the perturbation is removed [17]. The improvement of gait symmetry is possibly due to a patient’s damaged cerebral cortex that is unable to adjust his or her walking pattern to a natural environment such as overground [19]. However, since this approach stimulates symmetrical walking, it has been utilized as a rehabilitation option after stroke. As described previously, split-belt treadmill training applies a large perturbation with a predetermined ratio of two belts during walking. This causes difficulty in developing an individualized training plan. Investigating an intervention that is able to predict a person’s after-effects would be beneficial in creating a customized intervention method. Moreover, to individualize split-belt treadmill training and potentially predict its influence on step length asymmetry, it is necessary to control this specific gait parameter in real-time. Therefore, this study implements personalized split-belt speed ratios to improve control of participant step length and accomplish symmetric walking.

## Methods

### Participant selection

Eligibility of participants was determined based on stamina and lower-limb injury, as well as being 18 years of age or older. Only those who were free of lower limb injury for a minimum of 6 months prior to the start of the experiment were deemed eligible. Further, participants were informed they would need to walk for approximately 35 min on a treadmill with a weight added to one leg for most of the session. They were only scheduled if they were confident in their abilities to complete this task on two separate occasions. Upon arrival, participants were asked to fill out a survey to record their age, sex, height, weight, and weekly exercise level. All participants gave voluntary informed consent to participate in our study, which was approved by the University of South Florida’s Institutional Review Board. Participant characteristics are shown in Table 1.

**Table 1:** Participant self-reported characteristics

Subject	Gender	Age (years)	Height (in)	Weight (lbs)	^†^Exercise level	^^^BMI	Boot side
1	F	21	62	90	3	16.5	Right
2	F	19	64	120	2	20.6	Left
3	M	22	67	143	2	22.4	Left
4	M	25	65	150	NA	25.0	Left
5	M	18	70	165	3	23.7	Left
6	F	20	63	132	2	23.4	Right
7	F	19	61	107	4	20.2	Right
8	M	19	72	162	2	22.0	Left
9	F	22	63	128	5	22.7	Left
10	M	23	70	155	4	22.2	Left
11	M	20	66	202	1	32.6	Left
12	F	21	64	150	2	25.7	Left
13	F	20	65	168	2	28.0	Left
14	M	21	73	187	5	24.7	Left
		Average	Average	Average	Average	Average	
		20.71	66.07	147.07	2.85	23.54	
		SD	SD	SD	SD	SD	
		1.86	3.79	30.23	1.28	3.78	

*SD* standard deviation

^†^Exercise level: sedentary (1), light (2), moderate (3), active (4), very active (5), extra active (6)

^^^Body mass index (BMI) = Weight (lbs)/Height (in) * 703

### Data collection

**Fig. 1:**
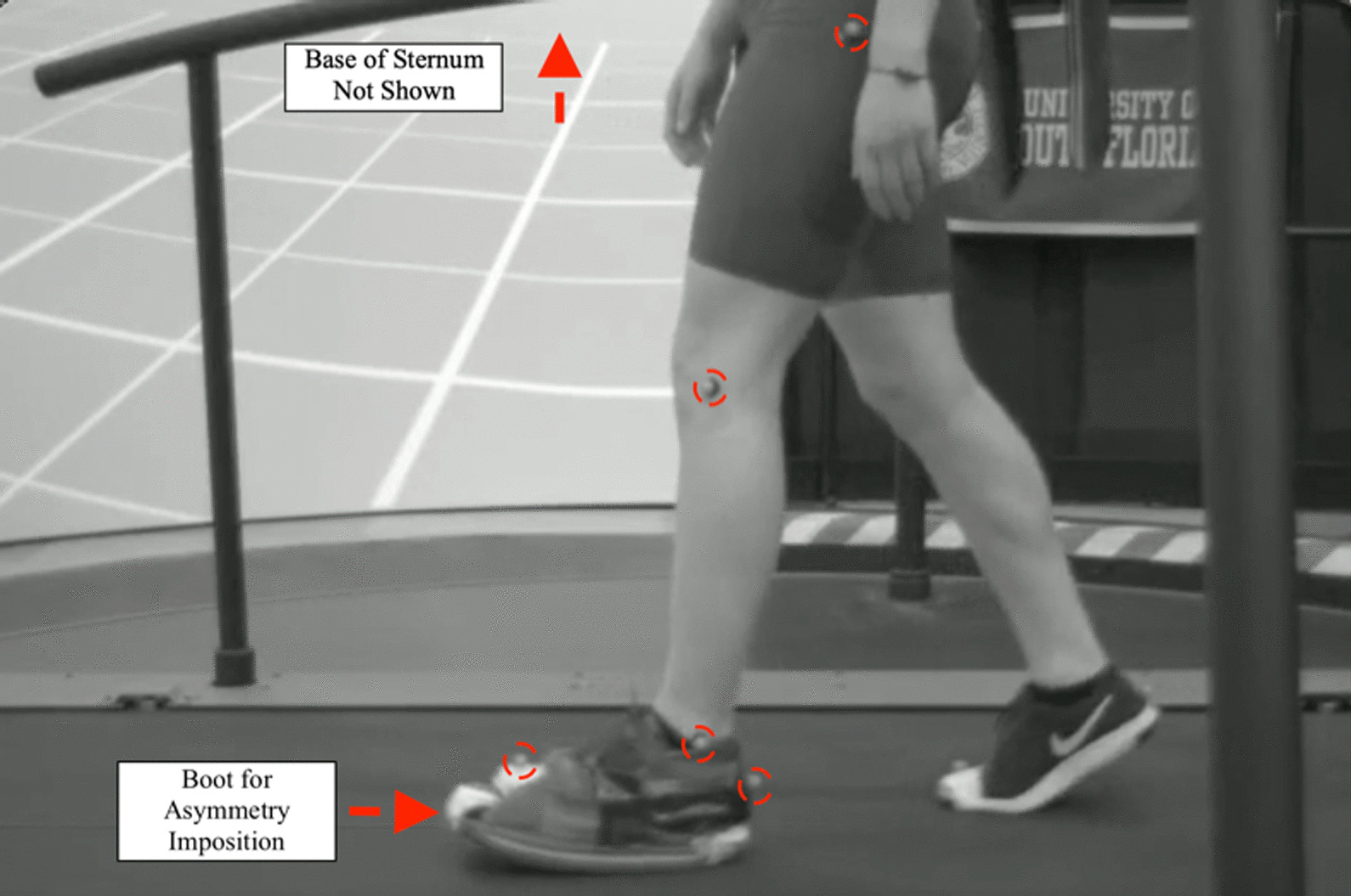
Marker setup for data collection. Right side has same placement, but not shown in this picture

The real-time feedback experiments were conducted on the Motek Computer Assisted Rehabilitation Environment (CAREN) system. A total of 10 Vicon cameras controlled by Nexus motion capture software (Version 2.1) were used. The kinematic data was recorded using 11 infrared markers (14 mm diameter), placed at the hips, knees (laterally), toes, ankles (laterally), heels, and sternum (as shown in Fig. 1). The split-belt treadmill capabilities of the CAREN system were controlled by a script written in the D-Flow control software. A real-time sorting algorithm was written in D-Flow to label and track the 11 markers. Offline analysis verified the accuracy of the measured parameters. In the first moment of the trial, prior to belt movement, markers were labeled based on the assumption that the participant was standing upright and still. Distances were calculated between every marker, and these distances, alongside standard gait assumptions, were used throughout the experiments to keep the markers appropriately sorted in real-time. Step lengths were calculated in real time by calculating the distance between heel markers for every step taken. A step was determined to be when the force plates received a reading of > 200 N (> 150 N in the case of the two lightest participants). The examiners conducting the experiments watched the marker motion in Vicon closely to verify the real-time calculations, ensuring that step length asymmetries were measured correctly.

Step length was measured in real-time for all trials conducted and used as the driving metric for split-belt adjustment in the feedback controller. Step length has been defined as the anterior–posterior distance from heel-strike of one foot to toe-off of the contralateral [10]. However, it has also been defined as the distance between heel locations upon heel-strike [21]. We opted to utilize the latter definition for the real-time algorithm. Thus, as participants walked, step length was recorded upon each heel strike as the difference between the anterior–posterior heel marker locations.

### Feedback controller

1 $$LL\mathrm{\%}=\frac{LSL-RSL}{ASL}*100\mathrm{\%}$$

$$LL{\%}=Left\, Leg\, Long\, Percentage; LSL=Left\, Step\, Length; RSL=Right\, Step\, Length; ASL=Average\, Step\, Length$$

Equation 1: Left-long (LL%) step length asymmetry

**Fig. 2:**
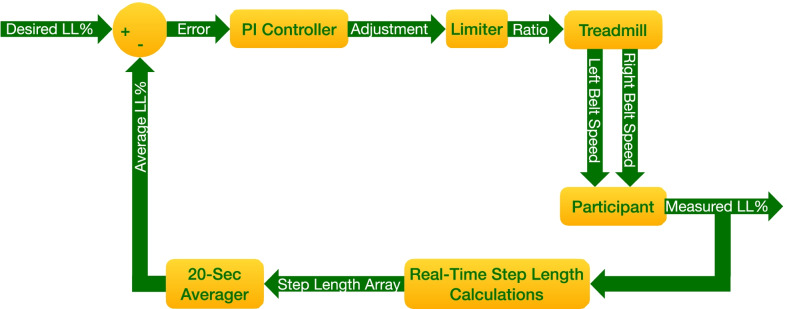
Block diagram of real-time feedback controller. LL% refers to “left long percentage” defined in Eq. 1

The purpose of the feedback controller (Fig. 2) was to enforce a specific step length asymmetry in participant gait. Therefore, the examiner conducting the experiment would input a desired LL% asymmetry (percentage by which left step length exceeds average step length). The LL% metric was used to measure both the magnitude of asymmetry as well as the direction, as positive values indicate a longer left step length and negative values indicate a longer right step length. Every 20 s, the left and right step lengths were averaged, and this average LL% was compared to the desired LL%. The error present was then sent to the proportional-integral (PI) controller, which utilized proportional and integral gains to calculate a necessary adjustment for each tread while keeping the average speed between the belts constant. This adjustment was processed by a limiter that only allowed 0.15 m/s to be added to/subtracted from each belt per adjustment. Further, this limiter enforced a maximum ratio of 3:1. The limiter sent the processed adjustment to the treadmill, which then modulated the left and right belt speeds. The participant’s step lengths were measured as they walked on this new ratio, and the process continued.

### Experimental protocol

Prior to the first training session, participants were instructed to walk on the tied-belt treadmill with an initial speed of 0.8 m/s. They were then asked to tell the examiner to speed up or slow down the treads until they felt comfortable. This speed was reported as the participant’s “comfortable walking speed.” The 11 markers mentioned above were then placed on the participant according to Fig. 1. For training session 1, participants walked for 3 min at tied-belt comfortable speed to gather an overall baseline. During this period, examiners were able to see the asymmetries, calculated using Eq. 1 every 20 s, but they were only saved and not used to adjust the tread ratios. After the baseline trial, a boot that added approximately 4.5 cm in height and weighed approximately 1 kg was added to the participant’s non-dominant leg. The dominant leg was determined by subject self-report. They were then asked to walk for 10 min under the same tied-belt condition. The last 3 min were averaged and considered the boot-baseline step length asymmetry (calculated using the last nine step length average measures).

**Fig. 3:**
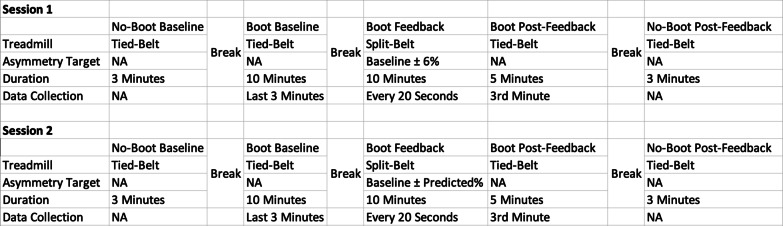
Experimental protocol in table format. Data collection row refers to timeframe in which data explicitly used in the study was collected

The measured asymmetry was then exaggerated by 6% (i.e., participant was driven further away from symmetry) by specifying the exaggerated asymmetry as the target into the split-belt control software. This exaggeration was used to evaluate the participant’s response to the tread-ratio feedback. Participants then walked for 10 min with the treads adjusting every 20 s according to the software to induce the desired target. The left and right treads started at the same speed but became asymmetric based upon the feedback controller’s output. Upon completion of the 10-min training session, the treads were returned to a tied-belt session without warning, and the participant’s post-intervention asymmetry was observed over a 5-min post-feedback period. The boot was then removed from the participant’s leg, and they were asked to walk for another 3 min with tied-belts. The protocol is outlined in Fig. 3.

The purpose of updating the tread ratios every 20 s was to continually push the participant to achieve the desired step length asymmetry. This continual push was intended to prevent the typical asymmetry reduction (sometimes referred to as a learning plateau) experienced by individuals walking on constant split-belt tread ratios [18]. As the participant plateaued, the software would recognize that the step length asymmetry was drifting away from the target and thus adjust the tread speeds accordingly so that the participant would continue to undergo training in the steeper portions of the adaptation curve. Once the step length was in the desired range, no more tread adjustments would occur. In the case that the starting step length is at the desired step length, no adjustment would occur, but this lack of adjustment is an atypical case.

The LL% (see Eq. 1) presented during the 3rd min of the boot post-feedback period was considered the usable post-intervention asymmetry. While split-belt interventions generally analyze the impact of the training period by evaluating step length asymmetry immediately following the return to tied-belt, this study performed the analysis during the 3rd min of tied-belt walking. The purpose of this delay was to evaluate the impact of the feedback training on a longer time scale. A linear relationship was then established from session one between 6% exaggeration and the level of improvement described by $LL\%\left(Boot\,Baseline\right)-LL\%(Post\,Intervention)$. The participant then returned for the second training session 1 week after the first. The only difference between the first and second sessions was the target asymmetry. After determining the boot-baseline in the second session, the value was entered into the linear relationship established based on the first session performance. The software would then output a predicted asymmetry exaggeration that would theoretically result in perfect post-intervention symmetry.

**Fig. 4:**
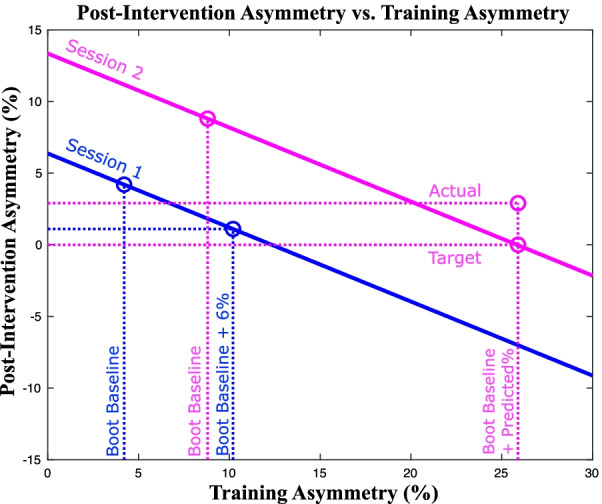
Linear model used for second-session target asymmetry prediction

Figure 4 demonstrates how this relationship is used. While the target asymmetry during the sessions was predicted by analyzing the relationship between baseline asymmetry exaggeration and post-intervention improvement over baseline, Fig. 4 relates actual asymmetry and actual post-intervention asymmetry (instead of exaggeration and improvement) to better visualize the linear theory. From this figure, it can be clearly seen that the slope of the line developed in session one (considered the evaluation session) can be used to predict a necessary target asymmetry for session two regardless of the participant’s baseline.

### Data analysis

**Fig. 5:**
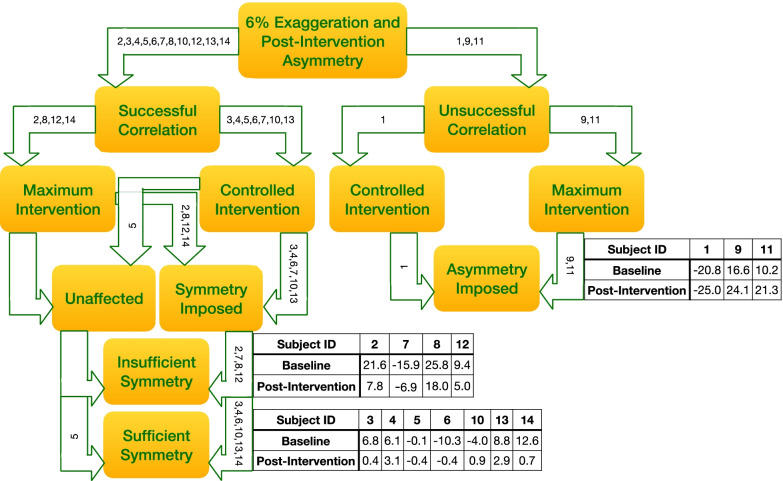
Outcome tree with categorized participant results. Baseline represents LL% with the boot on and post-intervention represents LL% during the post-feedback phase with the boot on (3rd min after returning to tied belts)

Participants can be classified into one of two categories based on the results of the first session: successful correlation and unsuccessful correlation (see first branch point of Fig. 5). Successful correlation refers to the magnitude of the post-intervention LL% being less than the magnitude of the boot-baseline LL%. Unsuccessful correlation, on the other hand, refers to the magnitude of the post-intervention LL% being greater than the magnitude of the boot-baseline LL%. It is well-known that using a split-belt intervention to exaggerate an existing asymmetry results in an after-effect of decreased asymmetry [15, 22]. Therefore, it was assumed that exaggerating the participant’s inherent boot-baseline asymmetry by 6% would result in a post-intervention LL% of lower magnitude. However, this was not always the case, and participants who experienced larger post-intervention asymmetries were considered part of the unsuccessful correlation group. There are two subcategories for participants that had an unsuccessful correlation (see right side of Fig. 5). Participants would either undergo a controlled intervention, where the predictor model suggests an attainable exaggeration, or a maximum intervention, where the predictor model suggests an unreasonably large exaggeration that results in a 3:1 ratio within the first 5 min. 3:1 was chosen as a maximum because a greater intervention was deemed unsafe. These categorization criteria are outlined in Eq. 2.

2 $$\begin{aligned} &\frac{LBS}{RBS}>3\, or\,\frac{RBS}{LBS}>3\, within\, first\, 5\, min \to Maximum\, intervention\\ &\frac{LBS}{RBS}<3\, and\,\frac{RBS}{LBS}<3\, for\, first\, 5\, min \to Controlled\, intervention\end{aligned}$$

$$LBS=Left\, Belt\, Speed; RBS=Right\, Belt\, Speed$$

Equation 2: Decision criteria for classification of maximum/controlled intervention branch

In either case, participants would theoretically have their initial asymmetry worsened. This is because the algorithm suggests decreasing the magnitude of their asymmetry or sending the participant to an LL% opposite in sign of their boot-baseline. This erroneous prediction is a direct result of the unsuccessful correlation previously mentioned. Therefore, participants in the unsuccessful correlation category undergo either controlled or maximum intervention, both of which should result in an imposed post-intervention asymmetry greater in magnitude than their baseline asymmetry (see bottom right of Fig. 5). Given that the feedback controller was unable to benefit these individuals because of the erroneous prediction, only the successful correlation category will be considered when evaluating the efficacy of the controlled intervention.

There are two subcategories for successful correlations, though the outcomes of these subcategories become more complicated (see left side of Fig. 5). Participants were placed in the controlled intervention subcategory or the maximum intervention subcategory by the same criteria seen in Eq. 2. In theory, both subcategories had the potential to either impose symmetry or produce no effect entirely. Participants were placed in the symmetry-imposed or the unaffected subcategories after the second session dependent upon the following criteria (Eq. 3).

3 $$\begin{aligned} &\left|LL{\%}\left(Post\,Intervention\right)\right|<\left|LL{\%}\left(Boot\,Baseline\right)\right|\to Symmetry \,imposed \\& \left|LL{\%}\left(Post\,Intervention\right)\right|\approx \left|LL{\%}\left(Boot\,Baseline\right)\right| \to {Unaffected}\end{aligned}$$

Equation 3: Decision criteria for classification of symmetry imposed or unaffected by training

Further, these participants were organized into groups of sufficient and insufficient symmetry (bottom left of Fig. 5). It has been previously suggested that gait parameters vary by approximately 3–4% in normal human gait [4, 12, 24]. While those figures are generally regarding forces in human gait rather than step lengths specifically, it is also known that abnormalities in gait are visible at a point somewhere between 5 and 13% asymmetry [3]. However, it has also been suggested that step length asymmetries in healthy individuals vary by only 3% [12]. Therefore, we opted to define sufficient symmetry as < 3.5%, which is well beneath the noticeable range and an average of the two values associated with normal human gait variation. Thus, participants were further categorized according to Eq. 4.

4 $$\begin{aligned} &\left|LL{\%}\left(Post\,Intervention\right)\right|\le 3.5{\%}\to Sufficient\, symmetry\, imposed\\ & \left|LL\mathrm{\%}\left(Post\,Intervention\right)\right|>3.5{\%}\to Insufficient\, symmetry\, imposed \end{aligned}$$

Equation 4: Decision criteria for classification after unaffected/symmetry-imposed branch

### Statistical analysis

A two-way repeated measures analysis of variance was conducted to analyze the effects of training. The independent variables were training with split-belt treadmill using PI feedback controller and session. The dependent variable was the amount of step length asymmetry change from baseline asymmetry with the boot on. All statistical tests were performed using IBM’s SPSS Statistics version 27. Mauchly’s Test of Sphericity and the Shapiro–Wilk test were checked. Post hoc tests with Bonferroni corrections were performed on results with statistical significance.

## Results

Statistical analysis revealed a significant main effect of training {F(1,13) = 5.869, p = 0.031}, indicating that split-belt treadmill training using the PI controller is effective in changing step length asymmetry between the baseline asymmetry (boot on) and the 3rd min of the post-feedback period. However, neither significant main effect of session nor interaction between training and session was found. Mauchly’s Test of Sphericity indicated that the assumption of sphericity had not been violated. Shapiro–Wilk test showed the assumption of normality had not been violated.

**Fig. 6:**
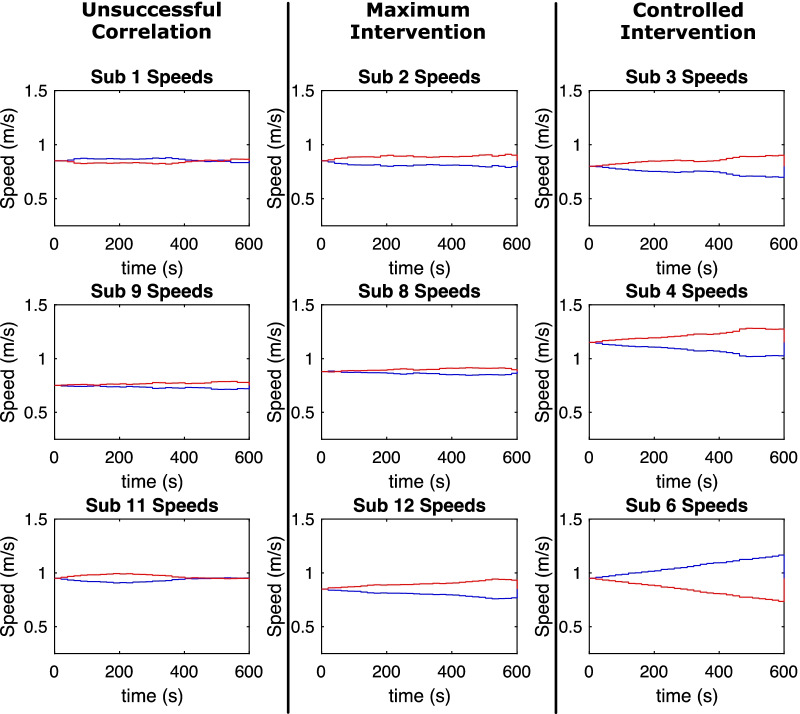
Session 1 belt speeds for participants in unsuccessful correlation (3/14), successful correlation—maximum intervention (4/14), and successful correlation—controlled intervention (7/14) categories (blue = left belt speed, red = right belt speed)

**Fig. 7:**
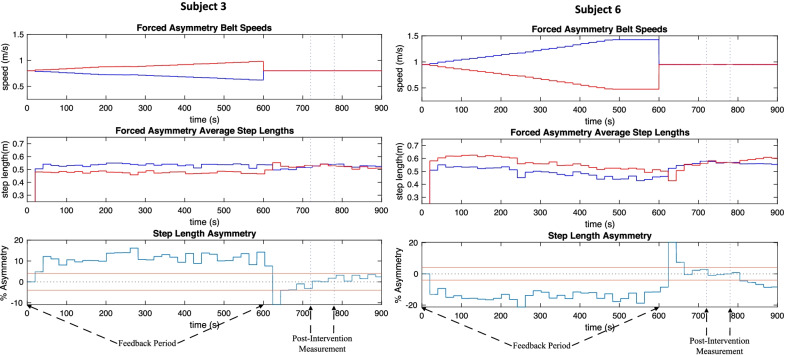
Session 2 controlled intervention (7/11) examples from the most responsive participants (for belt speed asymmetry graphs blue = left, red = right, for step length asymmetry graphs blue = step length asymmetry, red = sufficient symmetry border)

**Fig. 8:**
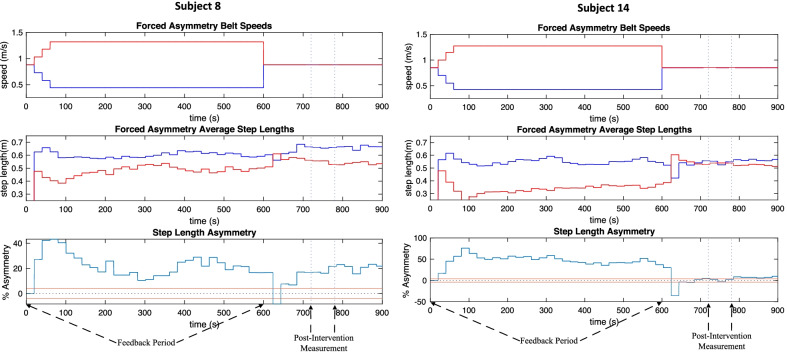
Session 2 maximum intervention (4/11) examples featuring the standard insufficient symmetry achievement (left) and the sufficient symmetry outlier (right) (for belt speed asymmetry graphs blue = left, red = right, for step length asymmetry graphs blue = step length asymmetry, red = sufficient symmetry border)

The primary goal of the first session was to enforce a specific step-length asymmetry upon the participants and collect the relationship between baseline asymmetry exaggeration and post-intervention asymmetry. The goal of the second session was to then enforce a predicted step-length asymmetry upon the participants and evaluate their proximity to symmetry in the post-intervention period. After the first session, 11 out of 14 (78%) participants were categorized as having a successful correlation. Three participants were categorized as having an unsuccessful correlation. In every unsuccessful correlation case, the split-belt speeds changed only a small amount and these three participants could be considered to have undergone no intervention (Fig. 6). Therefore, the successful correlation participant group (11/14) will be considered the entire data set for percentages stated in this section. Seven out of 11 participants (63.6%) underwent a controlled intervention in session two (see Fig. 7), while the remaining four participants (36.4%) underwent maximum intervention (see Fig. 8). All except one of the participants (90.9%) had symmetry imposed. The single participant that did not have symmetry imposed was unaffected because their boot-baseline was close to zero (− 0.11%), and thus the target asymmetry was essentially 0%. Seven participants (7/11, 63.6%) achieved sufficient symmetry, resulting in a reasonably high success rate for successfully correlated participants.

Six out of seven participants (85.7%) that underwent controlled intervention (7/11) achieved sufficient symmetry. On the other hand, only one out of four participants (25%) that underwent maximum intervention (4/11) achieved sufficient symmetry. Thus, the controlled intervention specifically yielded a very high success rate, while the maximum intervention was not consistently capable of inducing symmetry beneath 3.5% in magnitude. The session 2 details for the successfully correlated participants are outlined in Table 2.

**Table 2:** Baseline, target, real-time, and post-intervention asymmetries for session two of successfully correlated participants (11/14)

Sufficient symmetry achieved							
Subject	3	4	5	6	10	13	14
Baseline (%)	6.8	6.1	− 0.1	− 10.3	− 4	8.8	12.6
Target (%)	15.4	17.9	− 0.2	− 23.1	− 7.0	25.9	289.2
LiveEffect (%)	11.9	18.3	0.7	− 14.1	− 4.7	17.7	45.1
Error (%)	23.1	2.3	525.0	38.9	32.2	31.7	84.4
Post-intervention (%)	0.4	3.1	− 0.4	− 0.4	0.9	2.9	0.7

Insufficient symmetry achieved				
Subject	2	7	8	12
Baseline (%)	21.6	− 15.9	25.8	9.4
Target (%)	1462.2	− 20.7	259.6	127.7
LiveEffect (%)	34.0	− 24.8	18.6	9.9
Error (%)	97.7	20.2	92.8	92.2
Post-intervention (%)	7.8	− 6.9	18.0	5.0

## Discussion

This experiment demonstrated that the PI feedback controller was able to drive individuals to a specified asymmetry during training. Most of the participants responded favorably to the training such that only two sessions were required to generate and test a predictive model based on targeted step length asymmetry.

The different correlation categories had distinct differences in the outcomes of session one. Figure 6 demonstrates the differences between the belt-speeds of participants in the various categories during the session one training period. For participants in the unsuccessful correlation category (3/14, Fig. 6, left column), the speeds hardly changed during training session one when compared to participants in the successful correlation → controlled intervention category (7/11, Fig. 6, right column). Thus, the return to tied-belt resulted in no significant deviations from the baseline. In the case of participants in the successful correlation → maximum intervention category (4/11, Fig. 6, middle column), the belt speeds also hardly changed, though often more so than the unsuccessful correlation category. Currently, it is theorized that this issue arises out of an incorrect boot-baseline value. The participants in this category required little intervention to maintain the desired asymmetry, which was supposedly 6% greater than their baseline. However, the lack of required intervention leads one to believe that either the recorded baseline was inaccurate, or the participant was unresponsive to the intervention. Upon returning for the second session, these participants experienced large post-intervention asymmetries in the opposite direction of training (Fig. 5). Thus, an inaccurate baseline is the most likely explanation for the extremely small changes in belt speeds required to maintain the exaggerated asymmetry in session one. Thus, the importance of a true boot-baseline asymmetry is vital, and more research must be done to determine what constitutes “true boot-baseline asymmetry.”

### Categorized outcomes

There exists two groups of participants within the successful correlation category (11/14, see left branch of Fig. 5): those requiring a controlled intervention (7/11), and those requiring a maximum intervention (4/11). In some cases, participants displayed a weak correlation, meaning that a 6% exaggeration of asymmetry resulted in a very small improvement in post-intervention asymmetry over baseline asymmetry. These scenarios were often obvious, as the predictor model would suggest enormous exaggerations in the hundreds or thousands in session two. Clearly these values are unreachable, and so a maximum intervention is necessary. The split-belt was capped at a 3:1 ratio, and these participants reached this ratio within the first 5 min of training, often within the 1st min. In the maximum intervention cases (subjects 2, 8, 12, and 14), 75% of subjects did not reach sufficient symmetry (subjects 2, 8, and 12). However, only 14.3% of controlled interventions (subjects 3, 4, 5, 6, 7, 10, and 13) resulted in insufficient symmetry (subject 7). This study demonstrates that the real-time feedback controller is highly effective when a successful correlation exists and the controller does not reach its maximum value. More research should be done to understand why some participants fall into the unsuccessful correlation and maximum intervention categories. Given that participants who underwent 3:1 intervention exhibited some improvement in step length symmetry, it can be assumed that these participants were only capable of this limited level of improvement.

One participant from the controlled intervention category achieved insufficient symmetry (subject 7, baseline: − 15.9%, post-intervention: − 6.8%), and one participant from the maximum intervention category achieved sufficient symmetry (subject 14, baseline: 12.6%, post-intervention: 0.7%). While it was expected that the controlled intervention would not have a perfect success rate, the outcome of the maximum intervention outlier was surprising (see Fig. 8(right)). The precision of the post-intervention outcome in this case indicates that this participant was in fact capable of fully symmetric restoration, even though the baseline gathered from session 1 indicated otherwise.

The literature indicates that in the natural environment, people with stroke generalize the walking patterns adapted from a perturbed setting, e.g., split-belt treadmill [19]. However, gait training with a large perturbation appears to limit the generalization of the outcome of symmetrical walking. The use of gradual perturbations instead of sudden perturbations in post-stroke patients could make the intervention more generalizable [25]. The feedback controller studied here was, by nature, gradual in its perturbation imposition. Thus, the combination of gradual perturbations alongside targeted step length asymmetry could, in theory, have the potential to induce lasting and beneficial step length symmetries in persons who have had a stroke. Therefore, we recommend that future studies be conducted directly comparing existing step-length asymmetry correction strategies with the technique presented in this study in a post-stroke population. While this study has served as a proof-of-concept for the live feedback approach, further study will be needed to determine clinical relevance.

### Predictive model for session two

Evaluation of each participant’s first training session after witnessing the outcome of their second yields a clear pattern. The final categorization of each participant can be predicted with reasonable accuracy using the outcome of the first session. After session one, participants were characterized by the slope of the line relating asymmetry exaggeration and post-intervention improvement. For example, if the participant responded to the 6% exaggeration by displaying a post-intervention asymmetry 3% less than their baseline, the slope of their line would be − 0.5. Thus, positive slopes imply that asymmetry exaggeration worsens the post-intervention gait of the participant, which does not follow present findings [15, 22]. Therefore, participants can be sorted into successful or unsuccessful correlation groups immediately by the sign of the slope. Further, the magnitude of the slope is useful for predicting the outcome of session two. It was found that very small slopes correspond to very weak correlations, which often resulted in maximum interventions. On the other hand, larger slopes corresponded to stronger correlations, meaning participants had a higher likelihood of remaining within the controlled intervention criteria. The value 0.25 was chosen as a cutoff because it was half of the smallest slope found amongst the controlled intervention → sufficient symmetry imposed group.

This model was tested against the full study population and found to have an 85.7% success rate. The model failed for participants 7 and 14. Participant 7 was the only participant to maintain controlled intervention and not achieve sufficient symmetry, while participant 14 was the only participant to undergo maximum intervention and achieve sufficient symmetry. Thus, this model can identify outliers with proper adjustment to the cutoff slope value of 0.25 (the true value requires a larger data set for reliable prediction).

The ability to predict a patient’s response to the training session (session two) allows for informed decisions to be made upon completion of the evaluation session (session one). Not all participants respond to all forms of gait intervention, as has been demonstrated in rhythmic cueing experiments for step time rehabilitation [13]. Therefore, this predictive model could potentially give examiners the ability to identify possible split-belt training non-responders quickly. If after the first session it is likely that the participant will undergo a maximum intervention (small slope observed), it can be assumed that the individual either requires a larger exaggeration or can be classified as a non-responder. Thus, a second session of greater exaggeration and subsequent use of the predictive model would allow for classification of the participant. This approach would increase the efficiency of the training by flagging non-responders early in the rehabilitation process so that a more suitable training protocol may be suggested.

### Controller accuracy

Thus far, only baseline, target, and post-intervention metrics have been discussed. However, an important aspect of the real-time feedback intervention is the achieved asymmetry. The “real-time effect” was measured by averaging the LL% values over the last minute of session two training, just before the start of the cooldown period (tied-belts). This data is shown for all participants in the successful correlation category (11/14) in Table 2. In 85.7% of the successful correlation → controlled intervention (7/11) cases, the error between the real-time effect and the target calculated by Eq. 5 was < 40%, reaching 2.4% at best. This 40% value is rather large, and even still the results of these sessions were often very precise. Therefore, more research must be done regarding the fine tuning of the proportional and integral gains of the controller, as well as the step length averaging time frames (20 s in this study).

5 $$Error(\mathrm{\%})= \frac{Live-Target}{Target}*100\mathrm{\%}$$

Equation 5: Error calculation for comparing real-time effect to target asymmetry

Optimizing these values would likely result in tighter control of gait and allow for even more precise intervention. However, humans are often unpredictable in nature, and therefore achieving target asymmetries perfectly is unlikely. Ultimately, the most important aspect is continually fighting against the participant’s natural tendency to return to their baseline gait.

### Limitations

In the interest of time, participants were asked during the first session to walk for 10 min with the boot on and the baseline was considered the average of the last nine recordings (approximately the last 3 min). However, the standard deviations found within the recorded asymmetries amongst all the participants varies widely in these last nine recordings. Some participants with a large standard deviation fell into the successful correlation category, while some participants with a small standard deviation fell into the unsuccessful correlation category. Upon further evaluation of every participant’s baseline standard deviation, no clear distinction can be made between the baseline trials of participants who fell into the successful or unsuccessful categories. This leads to the assumption that the root cause of the errors in the first session are participant specific. Therefore, it may be necessary to conduct three sessions for participants in the unsuccessful category, the second of which is identical to the first, but with a larger exaggeration to force a response and gain a data point for the predictor model.

There are more parameters of interest in gait training than just step length asymmetry (e.g., stride length, step time, peak vertical force). The objective of this paper was to demonstrate that the feedback method could be a viable split-belt training approach; further understanding of the effects on these other parameters must also be investigated. An analysis of these other parameters was not included since this study intended to serve as a proof-of-concept for the manipulation of a given gait parameter using feedback. Thus, future studies will be conducted that analyze the effects of feedback training on parameters other than the controlling variable with the possibility of minimizing asymmetry in more than one parameter at a time. Further, studies should also be conducted to evaluate the effects of walking for 10 min with a weighted boot and no other perturbations. It is possible that the subjects’ post-intervention gait parameters could have been influenced not only by the training, but also by fatigue. Thus, to evaluate the effect of the feedback training alone, the effects of fatigue in this setting should be explored.

Another limitation of this study was the use of the moving 20-s time frame for step length averaging and subsequent belt adjustment. While this time frame was used in this study for the purpose of making real-time adjustments easily trackable, consideration should also be given to a step counting strategy. This strategy would employ the use of a counter that would trigger belt adjustments upon the completion of a certain number of steps rather than a certain period of time. This approach should be investigated further, as the time limitations of this study did not allow for in-depth research into the difference between time-dependent adjustments and count-dependent adjustments.

Finally, the methods used in this study are only one possible approach to testing the effects of feedback training on participants’ step length asymmetries. Enforcing a 6% exaggerated asymmetry allowed for some participants to avoid adapting altogether (unsuccessful correlation category). While it is unclear whether these individuals had inaccurate baseline asymmetries recorded during the baseline trials, this issue could be avoided altogether through a different methodology. Rather than setting a target asymmetry for the first session, future researchers may wish to impose a set tread ratio and record the asymmetry imposed. In this case, starting with a reasonable split-belt ratio could ensure that all individuals respond to session 1. Subsequent development of a relationship between step length asymmetry and the aforementioned ratio would allow for the prediction of a specific split-belt ratio needed to induce symmetry in much the same way as was done in this study. Future feedback studies should consider these alternative methods to circumvent some of the limitations present here.

## Conclusions

Whenever the real-time feedback controller was able to operate within a reasonable range, it succeeded in inducing sufficient symmetry in the post-intervention period with a success rate of 85.7% (7/11). However, this number does not represent the whole picture, as three participants did not allow the controller to stay within a reasonable range. Exactly 50% of those who participated in this study (7/14) exhibited a post-intervention asymmetry of < 3.5% after only two sessions. The stark contrast between the success rate of the controlled intervention and the success rate of the entire study shows that, while the controller is accurate, more intricate training plans are necessary to use the feedback effectively. Ultimately, the newfound ability to accurately control and predict gait parameters expands the horizon of rehabilitation engineering. It should, however, be noted that this study served as a proof-of-concept for the live feedback approach, and the clinical relevance must still be established through its application to post-stroke cases and direct comparison to existing therapies. Theoretically, this strategy may be applied to a wide variety of gait parameters to produce individualized therapies for optimized rehabilitation, though much more testing will be necessary to overcome the current limitations. Further, the ability to determine participants' training session outcomes (session two) from their baseline session outcomes (session one) with reasonable accuracy could provide great clinical benefit. In theory, several baseline sessions of varying real-time controlled therapies, such as split-belt and rhythmic cueing, could be performed to determine patient response to each of these interventions individually. These baseline sessions would allow for determination of which therapies the patient is sensitive to and which they are non-responsive to. When the controller was able to function, it did so with great success rates. Thus, the formation of a large dataset for a wide variety of therapies would give researchers the ability to quickly and efficiently pinpoint the necessary and highly effective interventions required by an individual.

## Data Availability

Please contact author for data requests.

## Abbreviations

CAREN: Computer Assisted Rehabilitation Environment
LL%: Left long percentage defined in Eq. 1
LSL/RSL: Left/right step length
ASL: Average step length
LBS/RBS: Left/right belt speed
